# Custom-Made Foot Orthoses as Non-Specific Chronic Low Back Pain and Pronated Foot Treatment

**DOI:** 10.3390/ijerph18136816

**Published:** 2021-06-25

**Authors:** Aurora Castro-Méndez, Inmaculada Concepción Palomo-Toucedo, Manuel Pabón-Carrasco, Javier Ramos-Ortega, Juan Antonio Díaz-Mancha, Lourdes María Fernández-Seguín

**Affiliations:** 1 Podiatry Department, University of Seville, C/Avicena s/n, 41009 Seville, Spain; ipalomo@us.es (I.C.P.-T.); mpabon2@us.es (M.P.-C.); jrortega@us.es (J.R.-O.); 2 Physiotherapy Department, University of Seville, C/Avicena s/n, 41009 Seville, Spain; jdm@us.es

**Keywords:** low back pain, foot, pronation, posture, custom-made foot orthoses

## Abstract

Excessive foot pronation has been reported as being related to chronic low back pain symptoms and risk factors in sports-specific pathologies. Compensating custom-made foot orthotics treatment has not been entirely explored as an effective therapy for chronic low back pain (CLBP). This study aims to observe the effects of custom-made foot orthoses, in subjects with foot pronation suffering from CLBP. A total of 101 patients with nonspecific CLBP and a pronated foot posture index (FPI) were studied. They were randomized in two groups: an experimental one (*n* = 53) used custom-made foot orthotics, and the control group (*n* = 48) were treated with non-biomechanical effect orthoses. The CLBP was measured using the Oswestry Disability Index (ODI) Questionnaire and a visual analogue scale (VAS), both for lower back pain. The symptoms were evaluated twice, at first when the subject was included in the study, and later, after 4 weeks of treatment. The analysis of outcomes showed a significant decrease in CLBP in the custom-made foot orthoses participants group (*p* < 0.001 ODI; *p* < 0.001 VAS). These findings suggest that controlling excessive foot pronation by using custom-made foot orthoses may significantly contribute to improving CLBP.

## 1. Introduction

Chronic low back pain (CLBP) is reported as a usual cause of disability in the world and higher prevalence in women, [1] probably due to sexual dimorphisms affecting the hip and the lower limb. Nonspecific CLBP is defined as a low back pain not attributable to a detectable or recognized specific pathology with a duration of >12 weeks, and therefore, it is difficult to improve this pathology. It is attributed physical and/or psychosocial factors without being related to musculoskeletal injuries, infectious, inflammatory, rheumatic, oncological or degenerative diseases. CLBP usually leads to functional disability and affects individuals’ daily lives [2,3].

Excessive foot pronation has been recognized as being linked to CLBP and may cause malalignment of the lower extremity (decreased range of ankle inversion, knee flexion range and higher knee and hip internal rotation) [4,5,6,7,8,9,10,11,12,13,14]. CLBP is considered a risk factor in sports-specific pathologies such as patellofemoral pain, medial tibial stress syndrome, etc. [15]. There is a wide variety of treatments for CLBP, and excessive pronation of the foot has been linked to CLBP [4,5,7]. The use of compensating custom-made orthotics for podiatric conditions has not been entirely explored as a useful therapy for CLBP.

Excessive foot pronation is associated with other factors, should be included in the evaluation of imbalances in the pelvis and lumbar spine area [6,9,10,13]. Foot pronation is evaluated with the foot posture index (FPI) [16] in relation to being a condition that causes kinematics changes in the lower extremity and increases the pelvic tilt, tension of the back muscles and lumbar hyperlordosis [17,18,19,20,21,22]. There is a lack of evidence on the effect of the custom-made foot orthoses in subjects suffering from CLBP and pronated foot to improve this syndrome by normalizing the foot posture [13,14,15,16,17,18,19,20,21].

The orthotics may improve the biomechanical response of the musculoskeletal system of the lower extremity in pronated foot patients during standing and walking; therefore, the aim of this research is to evaluate in both genders the effects of wearing custom-made foot orthoses on CLBP compared to the use of a placebo treatment for 4 weeks.

Our main hypothesis supports that customized foot orthoses as a treatment in people with excessive pronation of the foot improve CLBP.

## 2. Materials and Methods

### 2.1. Design

A double-blinded two-arm randomized controlled trial. The participants were randomly allocated to a custom-made foot orthoses intervention group or a placebo orthoses group.

The study was conducted in accordance with the Helsinki Declaration and approved by the Institutional Review Board (project code: 24-F-11).

This research report takes into account the CONSORT 2010 Statement guideline for randomized clinical trials [23]. 

The protocol was prospectively registered in clinicaltrials.gov with code NCT03996980. Date of registration: 25 June 2019.

### 2.2. Randomisation

Microsoft Excel’s randomization was used by an independent researcher to make the randomization sequence. The distribution of subjects in the control group and the experimental group was carried out in a similar way with a 1:1 ratio. An assistant who did not know the nature of the investigation safeguarded the sequence and distributed it in sealed envelopes.

### 2.3. Recruitment

All the subjects were recruited from the Podiatry Clinic Area of the University of Seville (ACP). The potential participants were patients who attended the ACP with any kind of foot pathology (nail and skin podiatric treatment or a biomechanical exploration) and had been previously diagnosed of nonspecific CLBP by a specialist or family physician. Participants were initially interviewed briefly to evaluated whether they would be suitable to include in the study. A qualified podiatrist carried out this initial assessment. Part of the sample was taken from a previous research that had an insufficient sample size to determine differences between genders (DOI:10.1177/0309364612471370). Based on a non-probabilistic convenience sampling, finally, 101 participants were recruited because 4 subjects were lost, 1 subject voluntarily left the study and we were unable to contact 3 subjects.

### 2.4. Inclusion Criteria

The inclusion criteria were women and men adults up to 65 years old, the presence of CLBP, and foot posture index pronated in one or both feet (henceforth, FPI) ≥+6 [16].

### 2.5. Exclusion Criteria

The exclusion criteria were serious illness, current participation in another research study, pregnancy, previous back or foot surgery, current treatment with foot orthoses, back pathology, or a leg length discrepancy >5 mm [24].

### 2.6. Procedure

All the 105 recruited subjects agreed to participate in the study and signed the informed consent. A podiatrist assessed the foot posture during the biomechanical assessment based on the six-item foot posture index (FPI ≥ +6) and regarding the compliance of the inclusion criteria. The FPI is a tool recommended by different authors as a validated method to identify podiatric disorders [16]. The FPI indicates a pronated foot position in a supported relaxed position when the FPI is equal to or superior to a +6 value and consists of six validated items measured in a standing relaxed position. The categories are: supinated foot: −1 to −12; neutral foot posture: 0 to +5 (neutral); pronated foot posture: +6 to +12.

Two types of interventions were made: the experimental group used a hand-made foot orthosis and the control one wore an insole without biomechanical effect; both treatments were applied to the patient once they were made (2 days after the exploration). The main researcher, who only evaluated, and the participants were blind to which group and kind of treatment was being studied. 

The primary outcome, CLBP, was evaluated twice using the Oswestry Disability Questionnaire [25] (ODI) and a 100 mm visual analogue scale [26] (VAS), both for lower back pain. The ODI explores the disability and the impact on ten daily activities due to the low back symptoms. Patients using the placebo insoles were finally treated according to their foot disorders. The first evaluation, or PRE-situation (PRE), was realized when the participants were included in the study, and the second one, or POST-situation (POST), when the treatment had been used for 4 weeks.

The recommendation of use of the treatment for both groups was equal: daily, at least for 8 h a day with their daily shoes. For the control group, custom-made foot orthoses were provided when the research was finished.

#### Fabrication of Foot Orthoses

**Figure 1:**
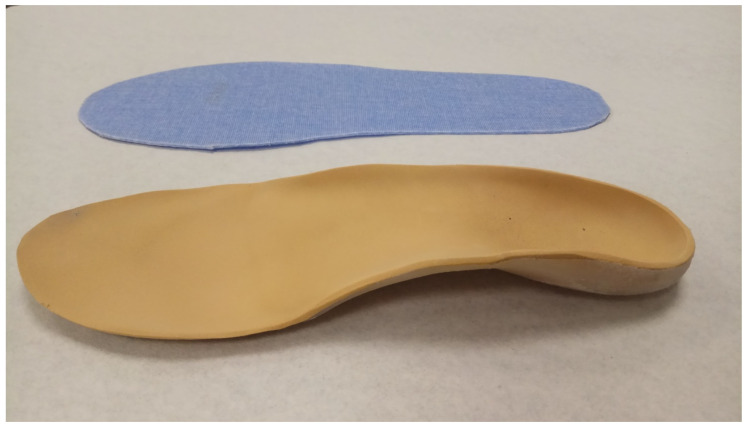
Foot orthoses.

Molds of both feet were obtained in a condition of all the participants’ feet under weight-bearing as they were manipulated to be placed in a neutral position. The positive mold of the plaster was used to make the custom orthoses in the experimental group. Polypropylene with 3 mm thickness (heated to 180 °C) was adapted from the rearfoot to the metatarsal parabola and covered with a 2 mm thick polyethylene foam 35 shore-A density upper. The control group used a flat insole. The placebo orthosis was made of polyester resin with a non-biomechanical effect (Figure 1).

### 2.7. Statistical Analysis

**Figure 2:**
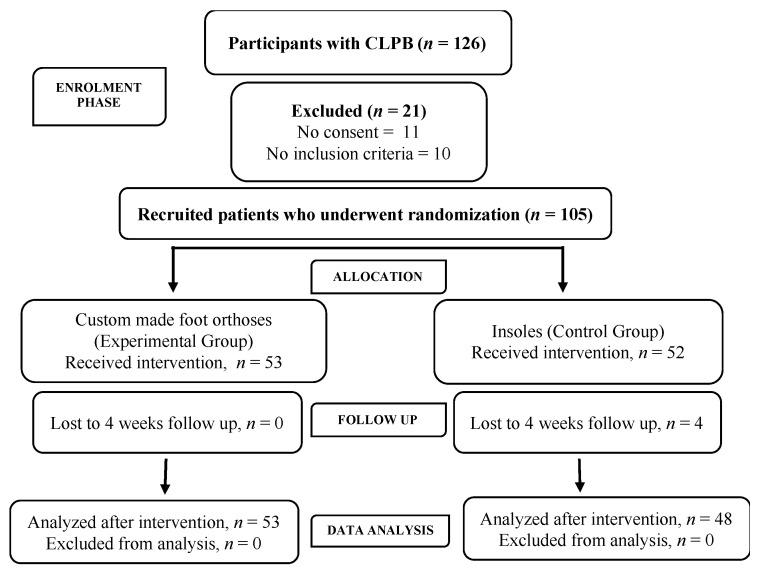
CONSORT flow diagram.

Based on a preliminary study [12], the sample size estimation was based on assuming a one-tailed hypothesis, a between-group allocation ratio of 1:1, a medium effect size (d = 0.6), an alpha value of 0.05, standard deviation of 17 points (minimal clinically important difference) and a desired power of 90% (Gpower 3.1.2, Kiel University, Germany). The sampling size necessary was 48 participants in each group. However, this was increased up to 105 subjects to compensate for potential participant dropout. Finally, a total of 101 participants finished the study (Figure 2).

The quantitative variables were expressed with means and standard deviations for each group: age, gender, BMI, FPI baseline. The Kolmogorov–Smirnov test was applied to determine the nature of the distribution of the sample data. We decided to use the non-parametric Mann–Whitney U test, as the normal distribution pattern was rejected. The PRE- and POST-situations of each group were compared using the Wilcoxon test. For the statistical analysis, the IBM SPSS Statistics 24.0. program was performed. An *p* value < 0.05 was set as statistically significant. The effect size was calculated as Rosenthal correlation. This parameter classifies the effect size as low if ≥0.20–0.40, moderate if ≥0.40–0.60, strong if 0.60–0.80 and very strong if ≥0.80. Differences were considered to be statistically significant if *p* < 0.05.

## 3. Results

The sample included 105 patients (54 females and 51 men). Finally, only 101 participants finished, because four subjects were lost.

There was a total of 101 participants (50.8% women and 48% males), aged between 18 and 65 years old (40.09, SD ± 15.22) with a body mass index (BMI) of 24.01, (SD ± 3.50). We reported 53.3% patients with a bilateral pronated foot and 46.5% with only one foot with FPI in pronation.

The groups were similar in age, gender, BMI, FPI, bilateral pronation, ankle range of movement (ROM) and at the moment of the initial exploration. Table 1 shows baseline data and initial distribution. Both groups were homogeneous in all variables (Mann–Whitney U test *p* > 0.05).

In the exploratory analysis, both groups seemed to be homogeneous as the dependent variable did not show differences between the groups measured on the two scales (VAS *p* = 0.54 and ODI *p* = 0.52). There were not significant differences between them, at the initial CLBP illness, respectively. A comparison was carried out between the experimental group at the beginning and at the end. In the same way, this took place with the control group. Statistical normality data analysis was carried out. Mann–Whitney U test were used with independent samples (Table 2).

Analysis for intent to treat was performed, finding similar results; so, for the experimental initial and end comparation, VAS *p* value = 0.0001 and ODI *p* = 0.0001. On the other hand, 41.7% of the sample improved more than 17 points on the ODI scale [27].

Therefore, Table 3 shows the changes in VAS and ODI variables in the experimental group were significant with a moderate effect size. However, nevertheless, in the control group, there were no significant differences following the use of the placebo insoles and the effect size was small. 

According to gender, the changes in VAS and ODI variables between groups (Table 4) showed there were significant differences after the treatment in both men and women.

## 4. Discussion

This study suggests exploring the relation between non-specific chronic low back symptoms in pronated foot subjects after a four-week intervention with custom-made foot orthoses or placebo foot orthoses in both genders. 

A double-blinded two-arm randomized controlled trial was carried out, measured with an ODI and a VAS scale. The results of the observation in subjects with FPI pronated foot and CLBP of the experimental treatment group showed significant statistical differences versus the control group for both genders. The measurements of patients after 4 weeks of intervention showed that there were significant differences in perceived CLBP before and after the use of the custom-made foot orthoses for the experimental group. In the control group, there were no significant differences from using the placebo. The symptoms’ improvement of the CLBP indicated significant differences in the intervention group (*p* < 0.001 ODI; *p* < 0.001 VAS).

In the preliminary research realized by us, similar results were obtained; a diminution of perceived CLBP was related to the use of the custom-made foot orthoses, as the control group did not show such an improvement. Actually, the distribution about gender was homogeneous, unlike the preliminary study (43 females and 2 males). Menz et al. stablished association between pronated foot function and CLBP only in women, but not in men [28]. Using the same type of intervention in all the patients, our results have not found differences between sex. Menz et al. explain their results by anthropometric differences of the pelvis in women with respect to men, with a greater static anterior pelvic tilt and dorsal inclination of the spine compared with men. However, we believe that the population age can influence on pain and activity limitation caused by CLBP. In the study of Menz et al., the participants ranged from 36 to 92 years old, with a mean of 64 years, whereas our sample was younger, with a range of 18–65, and a mean of 40 years old. Studies report that in women, levels of subjective health complaints were highest among those above 50 years of age [29]. In addition, degenerative bone disorders due to age can cause back pain in an older population [30], even more so in women due to menopause. Therefore, we think that factors related to age are more decisive in the results of low back pain treatment than anthropometric differences between men and women.

In a normal healthy population, pronated foot posture is the normal position at rest [31].

Excessive pronation in the rearfoot, unilateral or bilateral, may be related to the occurrence of pathological conditions of the lumbar spine [32,33]. Therefore, we hypothesized that wearing custom-made podiatry foot orthoses may contribute to normalizing the kinetic chain for the prevention or treatment of CLBP. This treatment influences foot stability in hyperpronated subjects, and improves the lower limb function and, consequently, the low back pain symptoms [12]. 

There is a lack of evidence to support the use of insoles as a therapy to improve the disability of CLBP [1,8,12,13,14,20]. According to some clinical observations, foot hyperpronation malalignment of the lower extremity and foot may induce structural and biomechanical deficits in the standing position and gait. Consequently, this situation may affect the sacral angle, pelvic inclination, and lumbar lordosis as a compensatory phenomenon, leading to non-specific chronic low back pain [7,17,18,19,20,34,35]. This situation may cause musculoskeletal imbalances of the lower limb and the occurrence of injuries, such as CLBP [4,7,10,36,37]. Due to all the compensations that excess pronation causes in the body of the subjects, foot function should be evaluated in clinical practice for patients with lower limb and low back pain [27].

Unilaterally, excessive pronation movement in a foot induces an internal rotation of the medial malleolus, pulling the tibia and femur internally. As a result, an ipsilateral pelvic tilt is presented and, consequently, during gait, a rotation of the lumbar vertebral. This situation alters the body’s kinetics, and hence can lead to the occurrence of CLBP [7,8,17,19,20].

A systematical review [20] evaluated the insole treatment for CLBP, including randomized controlled trials and crossover trials. The use of foot orthotics was compared with the placebo treatment. This research concluded that standard insoles are useless to prevent CLBP. For this reason and because the biomechanical movement of the two feet of a subject are different, we emphasize that the main results of this research are determined by the use of customized foot orthoses according to the individual requirement of each foot. 

### Limitations

One limitation is that we consider there to be a need for the time between the follow up to be longer. Another consists of the fact only excessive subtalar pronation was considered as a factor leading to CLBP. There was an improvement of the symptoms related with CLBP in the experimental group, but they did not disappear completely. This result may be similar to the same population, but age is a covariate that needs to be considered. Future studies are necessary.

## 5. Conclusions

The results suggest that subjects with pronated foot and idiopathic chronic low back pain treated with custom-made foot orthoses appear to experience a reduction in CLBP after a follow-up period of 4 weeks for both genders compared with subjects given a placebo treatment. 

## Figures and Tables

**Table 1:** Demographic data.

Variable	Total Sample = 101	Group	Group
		Experimental *n* = 53	Control *n* = 48
Gender male	48 (47.0%)	26 (49.10%)	22 (45.80%)
FPI BILATERAL PRONATED	54 (53.50%)	30 (56.60%)	24 (50.0%)
LIMITED ANKLE ROM FLEXION	46 (53.40%)	32 (60.4%)	22 (45.80%)
Age	40.09 ± 15.22	40.64 ± 15.46	39.48 ± 15.09
BMI	24.01 ± 3.50	23.7 ± 3.00	24.36 ± 3.95
FPI RIGHT FOOT	6.19 ± 1.50	5.98 ± 1.62	6.42 ± 1.33
FPI LEFT FOOT	6.28 ± 1.63	6.57 ± 1.21	5.96 ± 1.97

BMI: body mass index. FPI: foot posture index. ROM: range of motion.

**Table 2:** VAS and ODI scale for experimental and control group and valuation between PRE and POST situation.

VAS/ODI	Group Time Groups	Mean SD	95 IC	Median	Interquartile Range	*p* Value ^a^	Size Effect
VAS	**PRE**						
	Control group	6.50 ± 1.70	7.0–6.0	7.0	2		
	Experimental group	6.36 ± 1.70	6.8–5.9	6.0	2	0.505	
	**POST**						
	Control Group	6.54 ± 1.51	7.0–6.10	7.0	3		
	Experimental Group	3.02 ± 1.80	3.50–2.50	3.0	2	0.001	0.772
ODI	**PRE**						
	Control group	18.90 ± 9.40	21.50–16.30	18.0	12		
	Experimental group	20.50 ± 12.50	23.90–17.0	16.0	23	0.877	
	**POST**						
	Control Group	21.40 ± 8.40	23.80–19.10	20.0	13		
	Experimental Group	7.21 ± 6.20	8.90–5.20	6.0	10	0.001	0.771
VAS	**Control group**						
	between-group differences	−0.04 ± 1.88	0.5–0.57	0.0	2	0.941	
	**Experimental Group**						
	between-group differences	3.34 ± 2.41	4.0–2.70	3.0	3	0.001	0.647
ODI	**Control group**						
	between-group differences	−2.51 ± 10.42	0.38–5.40	0.0	10	0.150	
	**Experimental Group**						
	between-group differences	13.24 ± 12.73	16.80–9.70	8.0	22	0.001	0.593

^a^ Mann–Whitney U test; VAS: visual analogue scale; ODI: Oswestry Disability Questionnaire; SD: standard deviation; IC: confidence interval.

**Table 3:** Statistical significance and effect size for the perceived low back pain difference before and after using the treatment (experimental and placebo) in the experimental group and the control group.

Experimental Group	*p* Value ^b^	Size Effect
VAS PRE/POST	0.001	0.58
ODI PRE/POST	0.001	0.56
**Control Group**		
VAS PRE/POST	0.999	0.09
ODI PRE/POST	0.136	0.14

^b^ Wilcoxon test. VAS: visual analogue scale. ODI: Oswestry Disability Questionnaire.

**Table 4:** Statistical significance between groups according to gender.

Gender	VAS/ODI	Time/Change	Experimental Group Mean SD	Control Group Mean SD	*p* Value ^a^
MALE	VAS	PRE	6.50 ± 1.77	6.52 ± 1.44	0.926
		POST	2.81 ± 1.78	6.57 ± 1.40	<0.001
		CHANGE (PRE/POST)	3.69 ± 2.52	−0.04 ± 1.79	<0.001
	ODI	PRE	21.81 ± 12.89	17.17 ± 9.56	0.330
		POST	7.38 ± 7.32	20.78 ± 7.83	<0.001
		CHANGE (PRE/POST)	14.42 ± 13.86	−3.60 ± 10.87	<0.001
FAMALE	VAS	PRE	6.22 ± 1.92	6.48 ± 1.60	0.438
		POST	3.22 ± 1.84	6.52 ± 1.62	<0.001
		CHANGE (PRE/POST)	3.00 ± 2.30	−0.03 ± 1.99	<0.001
	ODI	PRE	19.15 ± 12.23	20.28 ± 9.17	0.445
		POST	7.04 ± 5.03	21.93 ± 8.84	<0.001
		CHANGE (PRE/POST)	12.11 ± 11.69	−1.65 ± 10.15	<0.001

^a^ Mann–Whitney U test; VAS: visual analogue scale; ODI: Oswestry Disability Questionnaire.
